# An ordered subset approach to including covariates in the transmission disequilibrium test

**DOI:** 10.1186/1753-6561-1-s1-s77

**Published:** 2007-12-18

**Authors:** Hervé Perdry, Brion S Maher, Marie-Claude Babron, Toby McHenry, Françoise Clerget-Darpoux, Mary L Marazita

**Affiliations:** 1 INSERM U535, BP 1000, Villejuif, 94817, France and Université Paris-Sud, IFR69, Villejuif, 94817, France; 2 Virginia Institute for Psychiatric and Behavioral Genetics, Virginia Commonwealth University, PO Box 980126, Richmond, Virginia 23298, USA; 3 Center for Craniofacial and Dental Genetics, Cellomics Suite 500, 100 Technology Drive, University of Pittsburgh School of Dental Medicine, Pittsburgh, Pennsylvania 15219, USA

## Abstract

Clinical heterogeneity of a disease may reflect an underlying genetic heterogeneity, which may hinder the detection of trait loci. Consequently, many statistical methods have been developed that allow for the detection of linkage and/or association signals in the presence of heterogeneity.

This report describes the work of two parallel investigations into similar approaches to ordered subset analysis, based on an observed covariate, in the framework of family-based association analysis using Genetic Analysis Workshop 15 simulated data.

With an appropriate choice of covariate, both approaches allow detection of two loci that are undetectable by the classical transmission-disequilibrium test. For a third locus, detectable by the classical transmission-disequilibrium test, a substantial increase of power of detection is shown.

## Background

For several diseases, one can suspect that clinical or environmental heterogeneity, expressed through covariates such that the age at onset (AAO) of the disease, its severity, or a measure of exposure to an environmental factor, is associated with genetic heterogeneity. This heterogeneity may hinder the detection of trait loci.

The ordered subset analysis (OSA) [1] was designed to perform linkage analysis based on the ordering of families on the values of a covariate (e.g., the average AAO of the affected members of a family). The aim of OSA is to test whether significant linkage can be observed in a subset consisting of the first families of this ordering, up to a certain rank that is not predefined.

The performance of OSA for detecting gene × environment interaction has been assessed [2], showing a substantial increase of power in many situations. Recently, Macgregor and colleagues [3] utilized a similar method in a proof-of-principle application with case-control genetic association analysis. Their approach was implemented in a scenario in which a quantitative trait related to the disease (e.g., severity of disease) was the covariate of interest. Although they did not investigate the efficiency of the approach under a variety of models, they nonetheless demonstrated it as a viable approach to conducting such analyses.

Herein, we introduce two slightly different approaches, inspired by OSA and by Spielman's transmission-disequilibrium test (TDT) [4], for family-based linkage and association testing. We apply these approaches to the Genetic Analysis Workshop 15 simulated data (Problem 3). The disease under study, mimicking the assumed rheumatoid arthritis model, results from a complex combination of genetic and environmental factors. The simulated model was known prior to the analyses.

The OSA-TDT approach is very similar to Hauser's approach, and aims at detecting linkage and association of a marker with the disease in a covariate-ordered subset of families. The order TDT (OTDT) approach (Perdry et al., unpublished data) is slightly different, and aims at the detection of genetic heterogeneity between the first and the last families of the ordering.

## Methods

### Data used

We used all 100 replicates of 1500 nuclear families. The ascertainment was based on the presence of at least two affected sibs. The true value of each covariate, including age at onset, severity, and IgM level, is given for all affected individuals. In each family, we chose as the index case the affected child who first appeared in the file; the studies were performed on trio families including the parent and the index.

The covariates used were AAO, disease severity, and a quantitative phenotype (IgM level). AAO depends on a latent variable that weighed the hazard, latent severity, and an independent random effect equally. Hazard and latent severity both depend on genotypes at different loci: all trait loci except G and H are involved in the hazard, whereas severity is determined by G and H only. In addition, Locus F influences disease risk directly through the IgM level. Thus, the IgM level is best conceptualized as an endophenotype.

For the OSA-TDT, both severity and IgM level were used separately as covariates, in ascending and descending ordering, in data from each of the 100 replicates. For the IgM covariate, we used a reduced data set, consisting of the first 250 families (due to the high overall power). For the severity covariate, we used both the reduced data set (250 families) and the full data set (1500 families). We selected two single-nucleotide polymorphisms (SNPs) nearest to Locus F for assessing power when IgM level was used as a covariate and two SNPs nearest Locus G and H when severity was used as covariate. In addition, we selected a group of SNPs unlinked with any simulated disease loci to demonstrate that the approach maintains the proper type I error rate. Additional details regarding selected SNPs can be found in Tables 1, 2, 3.

**Table 1:** Percent power to detect association using IgM as a covariate in TDT vs. OSA-TDT in 250 pedigrees

	No. replicates in which a significant effect was detected			
	
SNP^a^	*p *= 0.05	*p *= 0.01	*p *= 0.001	*p *= 0.0001
TDT without OSA				
SNP11_389	80	64	25	10
SNP11_390	4	1	0	0
				
OSA high				
SNP11_389	100	100	100	100
SNP11_390	6	2	0	0
				
OSA low				
SNP11_389	87	86	73	60
SNP11_390	9	1	0	0

^a^The SNPs tested were the SNPs nearest to Locus F.

**Table 2:** Percent power to detect association using severity as a covariate in TDT vs. OSA-TDT in 250 pedigrees

	No. replicates in which a significant effect was detected			
	
SNP^a^	*p *= 0.05	*p *= 0.01	*p *= 0.001	*p *= 0.0001
TDT without OSA				
SNP9_185	14	3	0	0
SNP9_186	2	0	0	0
SNP9_192	4	1	0	0
SNP9_193	5	0	0	0
				
OSA high				
SNP9_185	7	1	1	1
SNP9_186	11	4	1	0
SNP9_192	10	2	0	0
SNP9_193	7	1	0	0
				
OSA low				
SNP9_185	4	3	0	0
SNP9_186	13	2	0	0
SNP9_192	5	1	0	0
SNP9_193	3	0	0	0

^a^The SNPs tested were the SNPs nearest to Loci G and H.

**Table 3:** Percent power to detect association using severity as a covariate in TDT vs. OSA-TDT in 1500 pedigrees

	No. replicates in which a significant effect was detected			
	
SNP^a^	*p *= 0.05	*p *= 0.01	*p *= 0.001	*p *= 0.0001
TDT without OSA				
SNP9_185	2	0	0	0
SNP9_186	2	0	0	0
SNP9_192	6	2	0	0
SNP9_193	4	0	0	0
				
OSA high				
SNP9_185	22	8	2	0
SNP9_186	58	36	12	4
SNP9_192	20	5	2	0
SNP9_193	4	1	0	0
				
OSA low				
SNP9_185	11	2	0	0
SNP9_186	33	10	1	0
SNP9_192	6	2	1	0
SNP9_193	3	1	0	0

^a^The SNPs tested were the SNPs nearest to Loci G and H.

For the OTDT, we used only one covariate, namely the AAO, and on each replicate we used the full data set. We performed the classical TDT and the OTDT, focusing on the six non-HLA trait loci (A, B, E, F, G, H) that were given as candidates.

### Spielman's TDT

Both of our methods use the principle of Spielman's TDT [4]. This test is based on the observation of the transmission rate of a given allele at a bi-allelic marker locus by a heterozygous parent to an affected child; under the null hypothesis of no linkage or no association, this transmission rate is equal to 0.5. A value significantly different from 0.5 gives evidence of linkage and association of the disease with the marker.

### The OSA-TDT method

The OSA-TDT method is an application of OSA to Spielman's TDT. In the testing procedure, families are ranked in order based on the value of the quantitative covariate. The association test is performed on the initial subset, starting at either the highest or lowest values. Pedigrees are added sequentially, one at a time, until the entire sample is analyzed. At each sequential addition of cases, the association test is repeated. A given value *v *in the range of the values of the covariate divides the sample in two subsamples: the first subset of families of the ordering, up to the place where the value *v *is crossed, and the second subset, consisting of the last families of the ordering, starting from this very place. The smallest *p*-value obtained in any single ordered subset is the intermediate test statistic used for selecting the optimal value of *v*. Overall significance of the test is assessed via permutation. The permutation test is performed by repeating the process previously described in many (10,000) randomly ordered data sets. The minimum p-value from each randomly ordered data set is selected for inclusion in the null distribution against which the *p*-value from the covariate ordered subset is compared. The empirical *p*-value, representing the significance of covariate inclusion, is the proportion of *p*-values from the null distribution smaller than the *p*-value from the actual ordered subset. Analyses are repeated starting at both the highest and lowest values.

The permutation statistic is computed to test the null hypothesis H_0 _against the alternative hypothesis H_1_, defined as follows:

H_0_: There is no value of *v *for which there is evidence of linkage and association in the ordered subsample.

H_1_: For some *v*, there is evidence of linkage and association in the ordered subsample.

### The OTDT method

The OTDT is based on a very similar ordered-based division in subsamples, but the null and alternative hypothesis test are different. In the OTDT we are testing the specific hypothesis regarding the transmission rates of a given allele *M*_1 _at the locus *M *in the low and high subsample, designated here by *τ *and *ρ*. Our null hypothesis states that there is no difference between *τ *and *ρ*. We briefly describe the construction of our test, which is a log-likelihood ratio test.

A value *c *of the covariate is used to form two subsamples of families: *S*(*c*) (and *S'*(*c*)), where *S*(*c*) is the subset of families that has an associated covariate value less than *c*, and *S'*(*c*) is the subset that has an associated covariate value greater or equal to *c*. The general model has three parameters: a parameter *c *in the range of the values of the covariate, and two parameters *τ *and *ρ *in [0,1] that are the transmission rate of *M*_1_in *S*(*c*) and *S'*(*c*). The null and alternate hypothesis can be stated as follows:

H_0_: For every value of *c*, *τ *= *ρ*.

H_1_: There exists *c *such that *τ *and *ρ *are distinct.

Let *n*_1,*c *_and *n*_2,*c *_and *n'*_1,*c *_and *n'*_2,*c *_be the total number of transmissions of alleles *M*_1 _and *M*_2 _in the sample *S*(*c*) and *S'*(*c*), respectively. We let *n*_1 _= *n*_1,*c *_+ *n'*_1,*c *_and *n*_2 _= *n*_2,*c *_+ *n'*_2,*c*_. These are the number of transmissions of *M*_1 _and *M*_2 _in the whole sample of families.

The likelihood of *c*, *τ *and *ρ *can be written as $Z(c,\tau ,\rho )=\tau^{n_{1,c}}\cdot {\left(1-\tau \right)}^{n_{2,c}}\cdot \rho^{{n^{\prime }}_{1,c}}\cdot {\left(1-\rho \right)}^{{n^{\prime }}_{2,c}}$. Its maximum under the alternate hypothesis is $Z_{1}^{*}$ = *max*_*c*, *τ*, *ρ *_*Z*(*c*, *τ*, *ρ*). Similarly, under the null hypothesis, the maximum is $Z_{0}^{*}$ = *max*_*c*, *τ *_*Z*(*c*, *τ*, *τ*). Our log-likelihood ratio is now Ω = log($Z_{1}^{*}$/$Z_{0}^{*}$).

High values of Ω give support to H_1 _against H_0_. The *p*-value associated to the observed value of Ω is computed by a permutation procedure: the values of the covariate are randomly shuffled, and the values of Ω associated with these distributions of the covariate are computed to give an empirical distribution for Ω under the null hypothesis. The *p*-value is the proportion of Ω values from the null distribution greater than the observed value of Ω.

This test is symmetric. Its result does not depend on whether the ordering is ascending or descending. It aims primarily at the detection of heterogeneity. If the null hypothesis is rejected, then at least one of *τ *or *ρ *is different from 0.5, giving evidence for linkage and association with the disease locus in at least one of the subsamples.

## Results

### Results for OSA-TDT

We assessed the type I error rate of the OSA-TDT. The type I error rate is 1% at nominal alpha level of 0.01 across all of the SNPs. The average TDT *p*-value for SNPs unassociated with disease loci across covariates is 0.50. Thus, the permutation test exhibits the appropriate behavior.

The results for association between the SNPs closest to each disease locus and the RA phenotype conditioning on IgM level are summarized in Table 1. A SNP at Locus F (SNP11_389), the locus whose action works through the IgM level to influence RA risk, demonstrates a substantial increase in power to detect the association with RA risk. This increase is especially apparent at the lowest nominal alpha levels. In contrast, including disease severity as a covariate in OSA yields minimal power at the loci (G and H) that influence variation in disease severity in the 250 pedigree subsets (Table 2). However, there is an increase in power observed when the entire 1500 pedigree datasets are used (Table 3). There was essentially no power to detect all other disease loci, with a range of power of 2 to 11% per model when conditioning on the covariates.

### Results for OTDT

Table 4 displays the power of TDT and OTDT for Loci A, B, E, F, G, and H using severity as a covariate. The TDT shows a strong power of detection on Loci A, B, E, and F, whereas OTDT gives no or weak evidence of genetic heterogeneity in the sense described in the previous section.

**Table 4:** Percent power to detect association using severity as a covariate in TDT vs. OTDT in 1500 pedigrees

Locus	Chromosome	TDT	OTDT
A	16	97	21
B	8	83	6
E	18	100	52
F	11	100	37
G	9	6	100
H	9	4	100

However, the role of the Loci G and H is not detected by the TDT, but is detected by the OTDT in all replicates with a permuted *p*-value lower than 10^-4^. This illustrates genetic heterogeneity, reflected in the transmission rates of alleles at these loci. For each replicate, the test gives a critical value of the AAO: 31.3 and 31.8 years for Loci G and H, respectively (in both cases, the standard deviation is close to 7.3). Globally, the rate of transmission of one particular allele at these loci is significantly lower in families of patients with an AAO less than 31 years compared to that in families of patient with an AAO greater than 31 years.

## Discussion

Both methods, the OTDT and OSA-TDT, allowed detection of the involvement of Loci G and H in the simulated disease, using the AAO or the severity as a covariate. Indeed, we know from the answers that these loci are related to the severity, which is itself one of the elements used to determine the AAO. These loci are not related to the affection status, which is why they are undetectable by a classical TDT.

Locus F was also detected by the OSA-TDT method when families were conditioned on IgM level; this locus is indeed related to the IgM level, which directly influences the simulated RA risk; this influence explains why this locus is already detectable by a classical TDT. Nevertheless, we demonstrated dramatic increases in power when using small data sets. That is, the OSA-TDT method maintains very good power in conditions where the power of the TDT is low.

## Conclusion

The two ordering TDT methods allowed detection of two loci which are undetectable by the classical TDT, being unrelated to the affections status, but rather moderating its expression.

For a third locus, detectable by the classical TDT, a remarkable increase in the power for detection has been shown, thanks to the inclusion of an intermediate phenotype (the IgM level).

These ordering methods allow dealing with the particular situation in which a given covariate is suspected of reflecting heterogeneity of the disease. A distinct approach for testing heterogeneity, the predivided sample test [5], requires a prior determination of the potentially heterogeneous groups. An advantage of both OSA-TDT and OTDT is the removal of this requirement.

It appears that these ordering TDT methods are promising for detection of loci modulating the expression of a disease, or the value of an endophenotype.

## Competing interests

The author(s) declare that they have no competing interests.
